# Clients’ perceptions and satisfaction with HIV counselling and testing: A cross-sectional study in 56 HCT sites in South Africa

**DOI:** 10.4102/phcfm.v8i1.1173

**Published:** 2016-08-31

**Authors:** Gladys Matseke, Karl Peltzer, Neo Mohlabane

**Affiliations:** 1 HIV/AIDS/STIs and TB (HAST) Research Programme, Human Sciences Research Council, Pretoria, South Africa; 2 Department of Research & Innovation, University of Limpopo, Sovenga, South Africa; 3 ASEAN Institute for Health Development, Mahidol University, Salaya, Thailand

## Abstract

**Background:**

Client satisfaction serves as a predictor for acceptance of HIV counselling and testing (HCT) services. Therefore, the study of clients’ perception and satisfaction may offer insights on how to improve HCT programmes.

**Aim and setting:**

The aim of this study was to assess clients’ satisfaction with HCT as well as describe perceived barriers to and facilitators of HIV testing by HCT clients in South Africa.

**Methods:**

A cross-sectional survey was conducted through interviews with 498 clients purposefully selected at the end of an HCT visit at 56 HCT sites throughout the country.

**Results:**

All the 498 study participants had tested for HIV with 98.8% receiving their results. Most (88.2%) reported testing for HIV before. The vast majority (75.5%) of clients reported that they had decided to be tested for HIV by themselves. High levels of satisfaction with HCT service (89.8%), low levels (27.7%) of difficulty in making the decision to have an HIV test and high levels of perceived confidentiality (94.6%) of the HIV test results were reported in this study. The most cited perceived barrier to HIV testing was lack of awareness about the HCT service (98%), while staff attitudes (37%), confidentiality (29.6%) and privacy (23.6%) were perceived facilitators. In multivariate logistic regression, staff attitude was significantly associated with client satisfaction (*p* < 0.05).

**Conclusion:**

High levels of client satisfaction with HCT services were observed. Various barriers to and facilitators of – including staff attitude – HCT were identified which can help guide the improvement of HCT services in South Africa.

## Introduction

HIV counselling and testing (HCT) has become widely available in South Africa over the recent years, with more than 4500 public HCT sites.^1^ However, because of numerous factors related to clients and health systems, as indicated in various studies, uptake of HCT remains low in South Africa.^1^

Client-related factors that served as barriers to HIV testing included fear of diagnosis or doubts about HIV status,^2,3,4,5,6^ fear of disclosure of HIV test results^7^ and lack of awareness of testing sites.^8^ Distance to the HCT facility and lack of confidentiality in health facilities can also be barriers in accessing HIV testing services.^9^ Additional factors include fear of stigmatisation,^3,4,9,10^ transport difficulties^11^ and low risk perception.^9^ Peltzer et al.^9^ cited reasons such as knowing one’s status already, not understanding the group counselling process, long waiting times, disliking the counselling process and partner dynamics as obstacles to the utilisation of HIV testing services.

Sociodemographic factors, such as gender and age, have been cited as some of the reasons affecting utilisation of and access to HCT services.^12,13,14^ Men in some parts of Africa are under-represented in utilising health care facilities, including HIV testing, treatment and care.^13^ In South Africa, a review of testing rates from 2001 to 2006 in a public health facility showed that the rates of HIV testing were lower for men than non-pregnant women.^14^ Generally, it is acknowledged that men and the youth are hesitant to use public health facilities. However, according to Matovu et al.^11^ and Pettifor et al.,^15^ worries about confidentiality, health care workers’ attitude and cost and access to services are some of the reported barriers to health facility HCT usage among men and the youth.

Factors that encourage HIV testing include ‘the need to know one’s HIV status’.^16^ Knowing one’s HIV status can be achieved when HCT clients receive or return for their HIV test results at testing sites, but unfortunately this is not always the case as not all HCT clients receive or return for their results after HIV testing.^17,18,19,20^

Acceptance of HCT services is dependant on the clients’ satisfaction with the services provided.^20^ Client satisfaction in relation to health care is related to the clients’ decision pertaining to their choices in terms of health care plans, adherence to treatments and outcomes of the management.^21^ This model focuses on whether client expectations are ‘confirmed’ (and then satisfied) or disconfirmed (and then dissatisfied).^21^ Studies have shown that clients’ satisfaction with services is in turn predictive of factors such as male gender and educational level.^4,5^ Duration of the counselling service was associated with clients’ satisfaction.^22^ Long client waiting times and an unavailable health care provider may lead to client dissatisfaction.^23^ In South India, Papanna et al.^24^ highlighted that the cumulative percentage of client satisfaction with HCT was 60%. Dinku and Andargie^25^ assessed the quality of HCT in terms of client satisfaction comparing public and private health facilities. Reduced waiting time for test results was a determining factor for client satisfaction; compared with private facility clients those who tested in public facilities reported higher levels of satisfaction.^25^

The aim of this study was to assess clients’ satisfaction with HCT, as well as to describe barriers to and facilitators of HCT as perceived by HCT clients in South Africa.

## Research methods and design

### Study design

A cross-sectional survey was conducted through exit interviews of HCT clients in 56 HCT sites, in eight provinces in South Africa, over a period of 8 months from February to October 2012.

### Setting

The setting of the study comprised public and private HCT sites representing a variety of HCT service approaches in eight of nine provinces in South Africa. In a large number of public health facilities, ‘provider-initiated testing and counselling (PICT) also known as routine HIV testing (RT) and client-initiated counselling and testing (CICT) also known as VCT’ is offered in South Africa.^1^ HCT is also provided in the form of mobile services as well as in non-medical sites.^1^ ‘Options include HCT through community-based health services; stand-alone, mobile services, workplace services, home-based (home to home and index-patient models). These models can reach out to men, couples, children, adolescents, prisoners, migrant workers and other closed institutional settings where people will be unlikely to seek HCT on their own’.^26^

### Study population and sampling strategy

A total of 498 interviews with HCT users (minimum sample size needed 377) was conducted across all HCT sites. A two-stage sampling procedure was utilised. Firstly, one health district in each of the eight provinces was randomly chosen for inclusion in the study for the public HCT sites. Secondly, from the available lists of public and private HCT sites, four public and three private HCT sites were conveniently sampled per health district. Public, private and non-government organisations (NGOs) were chosen on the basis of service provision, for example, HCT service in a specific setting, using a specific model/approach (including PICT and community-based/mobile HCT services), and/or provision of services to a specific target population (such as men who have sex with men, commercial sex workers and youth), but was not sampled by the province.

A convenient sample of 10–15 HCT service users (aged 16 years or older) was targeted per HCT site, with the number of interviews being determined by the number of clients using HCT services during the site visit. Individual interviews were conducted with clients exiting the HCT services (sites) at the end of an HCT service visit. At the end of the HIV post-test counselling session, the counsellor who conducted post-test counselling informed the client about the study and referred the client to the study research staff.

### Data collection

Prior to exiting the HCT facility, the HCT client was interviewed with a questionnaire about the HCT service by external, trained research assistants. The questionnaire included questions relating to the participant’s demographics (three items), their HIV testing history (three items) and their HCT experiences during the visit (19 items). Questionnaire items were developed by the authors and derived from a literature review.^27,28,29,30^ The content validity of the questionnaire was reviewed by five experts, and the questionnaire was pilot tested for face validity and understanding with 15 HCT clients who were not part of the sample population.

### Data analysis

The data were analysed using the Statistical Package for Social Scientists (SPSS, Version 20.0). Descriptive statistical analysis was done to describe the study sample. Chi-square tests were used to investigate perceived facilitators and barriers of access to HCT across gender and type of facility. Multivariate logistic regression was conducted to determine associations between client satisfaction and its possible predictors for those variables that were considered to be significant (*p* < 0.05).

### Ethical considerations

The trained research staff from the Human Sciences Research Council introduced and explained the study and took informed consent from the patient. Interviews were conducted in a private area in or outside the HCT facility, using the dominant local language or in a language preferred by the participant. Ethics approval for the study protocol was obtained from the Human Sciences Research Council’s Ethics Committee (Application Number 9/19/08/09).

## Results

### Sample description

In all, 611 HCT users were approached, 498 agreed and 113 refused, and the response rate was 82.0%. A total of 498 HCT users were interviewed at post-test HIV counselling. The mean age of the HCT service users was 34 years (SD = 12.0), with most being females (62.2%) and 79.1% currently in a relationship. The majority of the HCT service users lived in urban or peri-urban areas, including informal settlements (59.4%), and attended government health services (68.6%). Majority of them were recruited from primary health care clinics (52.6%), followed by hospitals (21.4%) and outreach events (9.4%) (see Table 1).

**TABLE 1:** Sample characteristics of HCT users (*N* = 498).

Variable	*N* (%)
**Gender**	
Male	188 (37.8)
Female	310 (62.2)
**Age (years)**	
18–24	125 (25.2)
25–34	170 (34.3)
35–44	101 (20.4)
45–54	70 (14.1)
55 and older	30 (6.0)
**Currently in a relationship**	
Yes	391 (79.1)
No	103 (20.9)
**Geotype**	
Urban	158 (31.6)
Peri-urban	111 (22.2)
Informal settlement	28 (5.6)
Rural area	203 (40.6)
**Province**	
Western Cape	30 (6.0)
Eastern Cape	62 (12.4)
Northern Cape	39 (7.8)
Free State	48 (9.6)
KwaZulu-Natal	54 (10.8)
Gauteng	73 (14.6)
Mpumalanga	117 (23.4)
Limpopo	77 (15.4)
**Type of organisation**	
Government health service	343 (68.6)
NGO	145 (29.0)
Workplace	2 (0.4)
Private health service	10 (2.0)
**HCT type**	
Hospital	107 (21.4)
Primary health care clinic	263 (52.6)
Stand-alone	40 (8.0)
STI clinic	7 (1.4)
Mobile HCT service	15 (3.0)
Outreach	47 (9.4)
Community center	10 (2.0)
Workplace	10 (2.0)

*Source:* Author’s own work

HCT, HIV counselling and testing; NGO, non-government organisation.

### HIV testing history and experience

Overall, the majority (88.2%) of the HCT service users reported being tested for HIV before, with most (74.0%) reporting to have tested less than 12 months ago. Most (74.4%) reported to having tested for HIV at the same facility. Significantly more females (90.8%; *p* = 0.015) reported to having been tested for HIV at the same facility (78.4%; *p* = 0.008) before the day of the interview, compared with their male counterparts (83.8% and 67.3%, respectively). On the contrary, significantly more males had tested for HIV less than 12 months ago compared with females (80.5% vs. 70.5%; *p* = 0.043). More than two-thirds (68.7%) of the HCT users reported to have tested at HCT services at public health facilities, while only 31.3% had tested at HCT services at private health facilities (including NGOs and workplace). Significantly more males had tested for HIV at private health facilities compared with females (48.9% vs. 20.6%; *p* = 0.000), while more females had tested at public health services compared with males (79.4% vs. 51.1%).

Most (72.3%) of the clients reported that they did not perceive the decision to test for HIV as a difficult one. The majority (75.4%) of clients reported that they decided to test for HIV by themselves, 16.6% had been referred to an HIV test by a nurse, doctor or counsellor and 8.1% by either the partner, or a friend or a family member. An overwhelming majority (98.8%) reported that they had received their HIV test results. These findings did not vary by gender of the HCT client nor type of facility where the HCT service was provided.

Most HCT clients (98.6%) reported that the counsellor explained their results to them in a way that they were able to understand, with more females reporting this compared with males (100% vs. 96.3%; *p* = 0.001). The majority of the HCT clients reported being satisfied with the HIV testing. This finding did not vary by gender or type of facility. Majority of the HCT clients (78.5%) perceived that the amount of time spent talking to the counsellor was about right, with significantly more clients of private health facilities (85.8%) reporting this compared with public health facilities (74.9%). An overwhelming majority of the clients (98.6%) perceived that the length of time it took to receive their HIV test results was about right, with significantly more males and more HCT clients from private health facilities reporting this compared with their respective counterparts (72.6% vs. 64.4; *p* = 0.048, and 76% vs. 63.3%; *p* = 0.004). When asked about their perceptions regarding the confidentiality of their test results and discussion with the counsellor, the majority of the HCT clients believed that their HIV test results (94.6%) and discussion with counsellor (94.7%) would be kept confidential. These findings were significantly higher in the private compared with the public HCT sites and did not differ by gender (see Table 2).

**TABLE 2:** HIV testing history and HCT experience (*N* = 498).

Variable	All *N* (%)	Male *n* (%)	Female *n* (%)	Chi-square test *p*	Government health service *n* (%)	NGO / workplace / private *n* (%)	Chi-square test *p*
**Ever had an HIV test, before day of interview**							
Yes	434 (88.2)	155 (83.8)	277 (90.8)	0.015	291 (86.6)	143 (91.7)	0.068
No	58 (11.8)	30 (16.2)	28 (9.2)		45 (13.4)	13 (8.3)	
**Ever had an HIV test at same facility, before day of interview**							
Yes	328 (74.4)	107 (67.3)	221 (78.4)	0.008	241 (81.1)	89 (61.0)	< 0.001
No	113 (25.6)	52 (32.7)	61 (21.6)		56 (18.9)	57 (39.0)	
**How long ago did you have an HIV test?**							
< 12 months ago	331 (74.0)	128 (80.5)	203 (70.5)	0.043	214 (70.6)	119 (81.5)	0.022
1–2 years ago	47 (10.5)	15 (9.4)	32 (11.1)		33 (10.9)	14 (9.6)	
>2 years ago	69 (15.4)	16 (10.1)	53 (18.4)		56 (18.5)	13 (8.9)	
**Type of HCT facility**					-	-	-
Private (include NGO and workplace)	156 (31.3)	92 (48.9)	64 (20.6)	< 0.001			
Public health	342 (68.7)	96 (51.1)	246 (79.4)				
**Was it difficult to decide to get tested?**							
Yes	137 (27.7)	51 (27.4)	86 (28.1)	0.477	94 (27.7)	43 (27.7)	0.540
No	355 (72.2)	135 (72.6)	220 (71.9)		245 (73.2)	112 (73.2)	
**Decision for HIV testing**							
Decided by myself	373 (75.4)	132 (71)	241 (78)	0.212	251 (73.6)	124 (79.5)	0.127
Partner/friend/family member/other	40 (8.1)	18 (9.7)	22 (7.1)		26 (7.6)	14 (9.0)	
Doctor/nurse/counsellor	82 (16.6)	36 (19.4)	46 (14.9)		64 (18.8)	18 (11.5)	
**Were you given your HIV results?**							
Yes	489 (98.8)	183 (98.4)	304 (99.0)	0.408	334 (98.5)	155 (99.4)	0.387
No	6 (1.2)	3 (1.6)	3 (1.0)		5 (1.5)	1 (0.6)	
**Was your HIV test result explained to you in a way that you understood?**							
Yes	489 (98.6)	180 (96.3)	307 (100)	0.001	336 (99.1)	153 (97.5)	0.147
No	7 (1.4)	7 (3.7)	0 (0.0)		3 (0.9)	4 (2.5)	
**Perceived length of time spent talking to the counsellor**							
About right	386 (78.5)	155 (82.9)	231 (75.7)	0.145	254 (74.9)	133 (85.8)	0.004
Too short/too long	106 (21.5)	32 (17.1)	74 (24.3)		85 (25.1)	22 (14.2)	
**Were you satisfied with the HIV testing?**							
Yes	479 (98.6)	181 (98.9)	298 (98.3)	0.471	325 (97.9)	156 (100.0)	0.066
No	7 (1.4)	2 (1.1)	5 (1.7)		7 (2.1)	0 (0.0)	
**Perceived length of time to be tested and receive HIV test results**	326 (67.4)	130 (72.6)	195 (64.4)				
About right	138 (28.5)	46 (25.7)	91 (30.0)	0.048	209 (63.3)	117 (76.0)	0.004
Too short/too long	20 (4.1)	3 (1.7)	17 (5.6)		121 (36.7)	37 (24.0)	
**Do you believe your HIV test result will be kept confidential?**							
Yes	459 (94.6)	174 (95.1)	283 (94.3)	0.448	309 (93.4)	150 (97.4)	0.046
No	26 (5.4)	9 (4.9)	17 (5.7)		22 (6.6)	4 (2.6)	
**Do you believe that the discussion that you had with the counsellor will be kept confidential?**							
Yes	460 (94.7)	176 (96.2)	284 (93.7)	0.171	312 (93.4)	150 (97.4)	0.048
No	26 (5.3)	7 (3.8)	19 (6.3)		22 (6.6)	4 (2.6)	
**Are there things that you like about the HIV testing service at this site?**							
Yes	418 (89.5)	156 (90.7)	262 (88.8)	0.317	287 (86.7)	133 (96.4)	0.001
No	49 (10.5)	16 (9.3)	33 (11.2)		44 (13.3)	5 (3.6)	
**Are there things that you dislike about the HIV testing service at this site?**							
Yes	81 (17.0)	24 (13.6)	57 (19.0)	0.079	66 (20.1)	15 (9.9)	0.003
No	396 (83.0)	153 (86.4)	243 (81.0)		262 (79.9)	136 (90.1)	

*Source:* Author’s own work

HCT, HIV counselling and testing; NGO, non-government organisation.

### Perceived barriers and facilitators to HIV testing

Table 3 shows the factors that contribute as barriers of access to HCT services as perceived by HCT service users. The most cited factors perceived to be barriers to HIV testing were ‘people don’t know about the service’ (98%) and ‘concerns about being seen by other people using the site’ (50.8%). A few have mentioned the location of the site (15.8%) and lack of confidentiality (12%). Fewer participants mentioned the cost of the test (1.2%), the length of time it takes to be tested (5.4%), the length of time it takes to get the HIV test result (5.4%), lack of privacy (7.6%) and attitudes of staff (9.2%) as perceived barriers to HIV testing.

**TABLE 3:** Perceived barriers to using HIV testing services (*N* = 498).

Variable	All *N* (%)	Male *n* (%)^a^	Female *n* (%)^a^	Chi-square test *p*	Government health service *n* (%)^a^	NGO / workplace / private *n* (%)^a^	Chi-square test *p*
People don’t know about the service	49 (9.8)	30 (16.0)	18 (5.8)	< 0.001	20 (5.8)	29 (18.5)	< 0.001
The location of the site	79 (15.8)	30 (15.0)	49 (15.8)	0.964	47 (13.7)	32 (20.4)	0.040
The times that the site is open	31 (6.2)	8 (4.3)	21 (6.8)	0.245	19 (5.5)	12 (7.6)	0.237
The length of time it takes to be tested	27 (5.4)	12 (6.4)	15 (4.8)	0.461	23 (6.7)	04 (2.5)	0.039
The length of time it takes to get the HIV test result	13 (2.6)	7 (3.2)	13 (2.3)	0.359	12 (3.5)	01 (0.6)	0.050
The cost of the test	6 (1.2)	1 (0.5)	5 (1.6)	0.268	04 (1.2)	02 (1.3)	0.611
The lack of privacy	38 (7.6)	11 (5.9)	26 (8.4)	0.296	32 (9.3)	06 (3.8)	0.020
The lack of confidentiality	60 (12.0)	19 (10.1)	41 (13.2)	0.300	52 (15.2)	08 (5.1)	0.001
The attitudes of the staff	46 (9.2)	11 (5.9)	35 (11.3)	0.042	36 (10.5)	10 (6.4)	0.092
Concerns about being seen by other people using the site	254 (50.8)	90 (47.9)	163 (52.6)	0.308	170 (49.6)	84 (53.5)	0.235
Other (specify)	199 (39.8)	81 (43.1)	118 (38.1)	0.268	133 (38.8)	66 (42.0)	0.489

*Source:* Author’s own work

a , Owing to missing values, not all frequencies in the gender and type of facility columns add up to the total.

Significantly more males reported ‘people don’t know about the service’ (16%) as a perceived barrier to HIV testing than did females (5.8%; *p* < 0.001). Significantly more females reported attitudes of staff (11.3%) as a perceived barrier to HIV testing than did males (5.9%; *p* = 0.042). Significantly more HCT users from government health services reported lack of privacy (9.3%; *p* = 0.031) and lack of confidentiality (15.2%; *p* = 0.001) as perceived barriers to HIV testing than did HCT users from private health services/NGOs/workplace (3.8% and 5.1%, respectively). More HCT users from government health services reported attitudes of staff as a perceived barrier to HIV testing than did HCT users from private health services/NGOs/workplace (10.5% vs. 6.4%). However, this finding was not significant (*p* = 0.138) (see Table 3).

Table 4 indicates factors that facilitate the use of HCT services as perceived by HCT service users. The majority cited the attitudes of staff (37%), confidentiality (29.6%), people knowing about the service (29.2%), privacy (23.6%) as well as location of the site (23%). Almost 90% of the respondents mentioned that there are things they like about HIV testing service at the specific sites they visited (see Table 4).

**TABLE 4:** Perceived facilitators to HIV testing (*N* = 498).

Variable	Total *N* (%)	Gender		Type of Organisation	
			
		Male%	Female%	Government health service %	NGO / workplace / private %
The attitudes of the staff	185 (37.0)	45.4	54.6	62.2	37.8
People know about the service	146 (29.2)	33.1	66.9	64.4	35.6
The confidentiality	148 (29.6)	39.2	60.8	60.1	39.9
The privacy	118 (23.6)	39.8	60.2	66.9	33.1
Other	117 (23.4)	34.2	65.8	78.6	21.4
The location of the site	115 (23.0)	46.1	53.9	55.7	44.3
The times that the site is open	62 (12.4)	40.0	60.0	66.1	33.9
The length of time it takes to be tested	46 (9.2)	45.7	54.3	43.5	56.5
The length of time it takes to get the HIV test result	37 (7.4)	62.2	37.8	27.0	73.0
The cost of the test	30 (6.0)	53.3	46.7	43.3	56.7
Don’t mind being seen by other people using the site	56 (11.2)	33.9	66.1	60.7	39.3

*Source:* Author’s own work

### Client satisfaction with HIV counselling and testing

Regarding client satisfaction with HCT, the overwhelming majority (89.9%) reported being satisfied with the overall counselling experience (all six items measuring client satisfaction together). Almost all of the clients (99.2%) reported that they were satisfied with the type of person who counselled them, 97.6% were satisfied with the counselling they had received, 96.7% were satisfied with the consent process, 96.6% were satisfied with amount of privacy and 97.8% were satisfied with the treatment by the staff.

In multivariate logistic regression, staff attitude was significantly (*p* < 0.05) associated with overall client satisfaction (see Table 5).

**TABLE 5:** Associations with client satisfaction.

Characteristics	Satisfied with counselling and testing *N* (%)	Adjusted odds ratio (95% CI)^a^
**Gender**		
Males	154 (90.1)	1 (Ref.)
Females	257 (89.5)	0.98 (0.48–2.03)
**Age (years)**		
18–49	356 (89.7)	1 (Ref.)
50 and older	53 (89.8)	0.84 (0.33–2.21)
**Currently in a relationship**		
Yes	325 (89.5)	1 (Ref.)
No	86 (91.5)	1.25 (0.53–2.90)
**Type of area**		
Rural area	231 (46.2)	1 (Ref.)
Urban/Peri-urban	269 (53.8)	1.61 (0.77–3.33)
**Type of organisation**		

Government health service	285 (89.9)	1 (Ref.)

Private health service (includes NGO/workplace)	128 (89.5)	0.95 (0.43–2.09)
**Length of time spent talking to the counsellor**		
Too short/too long	83 (85.6)	1 (Ref.)
About right	328 (91.1)	1.26 (0.61–2.61)
**Length of time to be tested and receive HIV test results**		
Too short/too long	131 (88.5)	1 (Ref.)
About right	274 (91.0)	1.27 (0.57–2.85)
**Perceived barrier to HIV testing: staff attitude**		
Yes	35 (81.4)	1 (Ref.)
No	378 (90.6)	2.52 (1.05–6.07)*

*Source:* Author’s own work

a , Nagelkerke R Square = 0.032;

* , *p* < 0.05.

## Discussion

The study found high levels of satisfaction with HCT in a large sample of HCT clients across 56 HCT sites in almost all provinces in South Africa. This finding is in agreement with some previous studies, for example, in Ethiopia.^25^ The reported high levels of satisfaction with HCT in this study might have made it easy for the participants to make a decision to test for HIV; hence, the low rates of reported difficulty in deciding to test for HIV. Client satisfaction with HCT was significantly associated with having the perception that staff attitude is not a barrier to HIV testing. This finding is supported by the fact that a third of the study participants (37%) reported staff attitude as a facilitator to HIV testing while only a few (9.2%) reported this as a barrier.

Privacy of the counselling session is a huge feature in maintaining confidentiality. A large majority of the HCT clients in this study perceived that their HIV test results would be kept confidential. This might be an indication that clients had developed some level of trust with the clinic staff, specifically the counsellors. This was evidenced by the fact that 97% of the HCT clients were satisfied with the amount of privacy during the counselling session. As a result, traditional barriers relating to HCT, such as lack of confidentiality^2,31^ and poor standard of service,^32^ were minimal in this study.

The need to know one’s HIV status has been reported as one of the facilitators to HIV testing.^28^ Hence, almost all the participants in this study received their HIV test results, in contrast to some previous studies^17,18,20^ and understood the explanation given to them with regard to their HIV test results. The proportion of people who test and receive their results is among a set of reporting indicators on the implementation of the HCT programme.^1^ Albeit knowing one’s HIV status may be perceived as a facilitator to HIV testing, other factors related to client and service delivery that were perceived as facilitators to HIV testing among HCT clients in this study included attitudes of staff, people knowing about the service and location of the HIV testing site. This information can be utilised for improving staff attitudes and branding the HCT services.

Similarly, there were also numerous factors related to client and service delivery that were perceived as barriers to HIV testing in this study. Lack of awareness of HCT services at the sites was perceived as one of the major impediments to HIV testing. Furthermore, just over half of the HCT clients in this study reported ‘concerns about being seen by other people using the site’ as a barrier to HIV testing. This result confirms the finding by Bwambale et al.^33^ in a study that assessed HCT uptake among men in Uganda pertaining to location of the testing site. Men were less likely to access HIV testing services; factors related to fear of being recognised by familiar people at local clinics as well as confidentiality were cited as barriers to HIV testing.^33^ In addition, the finding reported in the present study may be an indication that the stigma related to HIV may still be a significant barrier to HIV testing among communities. Inadequate counselling space and shortage of counselling/clinic staff has always posed a threat to service delivery in South African public health facilities.^9^ Similar problems are also evident in this study based on the perceptions of HCT users.

The finding that fewer males compared with females in this study reported testing for HIV confirms the commonly held view that men often have less contact with public health facilities compared with women. A study conducted by Bwambale et al.^33^ reported similar findings about men’s attendance of HCT services. This subsequently reduces their opportunities for accessing and utilising HIV prevention, care and treatment services.^13^ However, the use of mobile HCT has proven to be effective in reaching and increasing HCT uptake among men.^32^ The same pattern is evident in this study where all the 15 participants (3%) who used mobile HCT services were men. Moreover, more men than women utilised non-public health facilities (e.g. NGOs) for HIV testing in this study. This might suggest that men find the services at non-public health clinics more friendly and acceptable as compared with those in public health clinics.

Despite the reported perceived barriers to HIV testing, it is motivating to note that most participants in this study made the decision to test for HIV by themselves. However, the low percentage of clinic staff who encourage participants to test for HIV during their visits at the site is of concern, as it means that those who are uncertain about testing or knowing their HIV status may leave the site without being tested, owing to lack of encouragement from the counselling or clinic staff. The government has recently recommended routine provider-initiated HIV testing and counselling (PITC) to address the low uptake of HCT.^11^ However, its implementation has been a challenge in under-resourced public health facilities with health care workers citing factors such as high patient loads as deterrents to implementation of PITC.^34^

### Limitations

This was a health facility–based survey and relied on self-report by participants. This may have led to reporting bias. Further, the sampling strategy was not representative and therefore the results cannot be generalised to the whole country.

## Conclusion

High levels of client satisfaction with HCT services were observed. Various HCT barriers such as awareness about the HCT service and HCT facilitators, staff attitude, confidentiality and privacy were identified which can help guide the improvement of HCT services in South Africa.

## References

[1] National Department of Health. South African national HIV counselling and testing (HCT) policy guidelines, 2010 Pretoria: Department of Health2010.

[2] Mphaya J, Roos J, Ehlers V Factors motivating young people in Malawi to go for voluntary counselling and testing for HIV. Afr J Nurs Midwifery. 2008;10(1):59–77.

[3] Meiberg AE, Bos A, Onya HE, Schaalma HP Fear of stigmatization as barrier to voluntary HIV counselling and testing in South Africa. East Afr J Public Health. 2008;5(2):49–54. 19024410

[4] Day JH, Miyamura K, Grant AD, et al. Attitudes to HIV voluntary counselling and testing mineworkers in South Africa: Will availability of antiretroviral therapy encourage testing? AIDS Care. 2003;15(5):665–672. http://dx.doi.org/10.1080/0954012030001595140 12959817 10.1080/0954012030001595140

[5] Kebaabetswe P Barriers to participation in the prevention of mother-to-child HIV transmission program in Gaborone, Botswana a qualitative approach. AIDS Care. 2007;19(3):355–360. http://dx.doi.org/10.1080/09540120600942407 17453569 10.1080/09540120600942407

[6] Ginwalla S, Grant A, Day J, et al. Use of UNAIDS tools to evaluate HIV voluntary counselling and testing services for mine workers in South Africa. AIDS Care. 2002;14(5):707–726. http://dx.doi.org/10.1080/0954012021000005533 12419119 10.1080/0954012021000005533

[7] van Dyk AC, van Dyk PJ ‘To know or not to know’: Service-related barriers to voluntary HIV counseling and testing (VCT) in South Africa. Curationis 2003;26(1):4–10. http://dx.doi.org/10.4102/curationis.v26i1.1289 10.4102/curationis.v26i1.1289 14509113

[8] Tenkorang EY, Owusu GA Correlates of HIV testing among women in Ghana: Some evidence from the Demographic and Health Surveys. AIDS Care. 2010;22(3):296–307. http://dx.doi.org/10.1080/09540120903193716 20390509 10.1080/09540120903193716

[9] Peltzer K, Matseke G, Mzolo T, Majaja M Determinants of knowledge of HIV status in South Africa: Results from a population-based HIV survey. BMC Public Health 2009;9(1):174 http://dx.doi.org/10.1186/1471-2458-9-174 10.1186/1471-2458-9-174 PMC2700104 19500373

[10] Kalichman SC, Simbayi LC HIV testing attitudes, AIDS stigma, and voluntary HIV counselling and testing in a black township in Cape Town, South Africa Sex Transm Infect 2003;79(6):442–447. http://dx.doi.org/10.1136/sti.79.6.442 10.1136/sti.79.6.442 PMC1744787 14663117

[11] Matovu JK, Makumbi FE Expanding access to voluntary HIV counselling and testing in sub-Saharan Africa: Alternative approaches for improving uptake, 2001–2007. Trop Med Intern Health. 2007;12(11):1315–1322. http://dx.doi.org/10.1111/j.1365-3156.2007.01923.x 10.1111/j.1365-3156.2007.01923.x 17949401

[12] Mills EJ, Beyrer C, Birungi J, Dybul MR Engaging men in prevention and care for HIV/AIDS in Africa. PLoS Med 2012;9(2):e1001167 http://dx.doi.org/10.1371/journal.pmed.1001167 10.1371/journal.pmed.1001167 PMC3274499 22346735

[13] April MD, Walensky RP, Chang Y, et al. HIV testing rates and outcomes in a South African community, 2001–2006: Implications for expanded screening policies. J Acquir Immune Defic Syndr. 2009;51(3):310 http://dx.doi.org/10.1097/QAI.0b013e3181a248e6 19582895 10.1097/qai.0b013e3181a248e6 PMC3209660

[14] Pettifor A, MacPhail C, Rees H, Cohen M HIV and sexual behavior among young people: The South African paradox. Sex Transm Dis 2008;35(10):843–844. http://dx.doi.org/10.1097/OLQ.0b013e31818318c0 10.1097/OLQ.0b013e31818318c0 18716569

[15] Killewo J, Kwesigabo G, Comoro C, et al. Acceptability of voluntary HIV testing with counselling in a rural village in Kagera, Tanzania. AIDS Care. 1998;10(4):431–439. http://dx.doi.org/10.1080/09540129850123966 9828963 10.1080/09540129850123966

[16] Kilewo C, Massawe A, Lyamuya E, et al. HIV counseling and testing of pregnant women in sub-Saharan Africa: Experiences from a study on prevention of mother-to-child HIV-1 transmission in Dar es Salaam, Tanzania. J Acquired Immune Defic Syndr. 2001;28(5):458–462. http://dx.doi.org/10.1097/00042560-200112150-00009 11744835 10.1097/00042560-200112150-00009

[17] Kiarie J, Nduati R, Koigi K, Musia J, John G HIV-1 testing in pregnancy: Acceptability and correlates of return for test results. AIDS. 2000;14(10):1468–1470. http://dx.doi.org/10.1097/00002030-200007070-00030 10930172 10.1097/00002030-200007070-00030

[18] Cartoux M, Msellati P, Meda N, et al. Attitude of pregnant women towards HIV testing in Abidjan, Côte d’Ivoire and Bobo-Dioulasso, Burkina Faso. AIDS. 1998;12(17):2337–2344. http://dx.doi.org/10.1097/00002030-199817000-00016 9863877 10.1097/00002030-199817000-00016 PMC4710787

[19] Fylkesnes K, Siziya S A randomized trial on acceptability of voluntary HIV counselling and testing. Trop Med Int Health. 2004;9(5):566–572. http://dx.doi.org/10.1111/j.1365-3156.2004.01231.x 15117300 10.1111/j.1365-3156.2004.01231.x

[20] Gupta M Profile of clients tested HIV positive in a voluntary counselling and testing center of a district hospital, Udupi. Indian J Community Med. 2009;34(3):223–225. http://dx.doi.org/10.4103/0970-0218.55288 20049300 10.4103/0970-0218.55288 PMC2800902

[21] Ismail H, Ali A Pregnant women’s satisfaction and comprehension level of information given during HIV Counseling and Testing for PMTCT in public health facilities in Addis Ababa. Ethiopian J Health Dev. 2011;25(2):126–134.

[22] The Bureau of Health Promotion: Evaluation of Voluntary Counselling and Testing in the National Prevention of Mother to Child Transmission Programme [serial online] 2000 [cited 2015 Jul 15]. Available from: http://www.searo.who.int/LinkFiles/Evaluation_of_Voluntary_Counselling_complete.pdf

[23] Rashmi, Vijaykumar B Assessment of the quality of service given by health care provider about tuberculosis in RNTCP. Indian J Community Med. 2010;35(2):368–369. http://dx.doi.org/10.4103/0970-0218.66870 20922132 10.4103/0970-0218.66870 PMC2940211

[24] Papanna MK, Kumar P, Shetty A, Kamath A, Bhaskaran U, Saddichha S Client satisfaction with HIV counseling services: A cross-sectional study from South India. J Int Assoc Provid AIDS Care. 2013;12(3):169–172. http://dx.doi.org/10.1177/1545109712444754 22588314 10.1177/1545109712444754

[25] Dinku F, Andargie G Assessment of Voluntary Counseling and Testing (VCT) service quality in terms of client satisfaction: A comparative study between public and private health institutions in Addis Ababa, Ethiopia. Sci J Clin Med. 2013;2(1):1–7. http://dx.doi.org/10.11648/j.sjcm.20130201.11

[26] Department of Health. National HIV counselling and testing policy guidelines, 2015 Pretoria: Department of Health; 2015.

[27] UNAIDS. Tools for evaluating HIV voluntary counselling and testing Geneva, Switzerland: UNAIDS; 2000.

[28] Richter L, Durrheim K, Griesel D, Solomon V, van Rooyen H Evaluation of HIV/AIDS Counselling in South Africa. Contract report submitted to the Department of Health. Pietermaritzburg: School of Psychology, University of Natal; 1999.

[29] Magongo B, Magwaza S, Mathambo V, Makhanya N National Report on the Assessment of the Public Sector’s Voluntary Counselling and Testing Programme. Durban: Health Systems Trust; 2002.

[30] Ndhlovu L, Searle C, Miller R, Fisher A, Snyman E, Sloan N Reproductive Health Services in KwaZulu Natal, South Africa: A Situation Analysis Study Focusing on HIV/AIDS Services. New York: The Population Council; 2003.

[31] South African National AIDS Council National Strategic Plan on HIV, STIs and TB, 2012–2016. Pretoria: Department of Health; 2012.

[32] De Cock KM, Mbori-Ngacha D, Marum E Shadow on the continent: Public health and HIV/AIDS in Africa in the 21st century. Lancet. 2002;360(9326):67–72. http://dx.doi.org/10.1016/S0140-6736(02)09337-6 12114058 10.1016/S0140-6736(02)09337-6

[33] Bwambale FM, Ssali SN, Byaruhanga S, Kalyango JN, Karamagi CA Voluntary HIV counselling and testing among men in rural western Uganda: Implications for HIV prevention. BMC Public Health. 2008;8(1):1 http://dx.doi.org/10.1186/1471-2458-8-263 18664301 10.1186/1471-2458-8-263 PMC2529297

[34] Leon N, Colvin CJ, Lewin S, Mathews C Provider-initiated testing and counselling for HIV: From debate to implementation. S Afr Med J. 2010;100(4):220–221. http://dx.doi.org/10.7196/SAMJ.4043 20459964 10.7196/samj.4043

